# Decisions to Practice in Rural Areas Among Mental Health Care Professionals

**DOI:** 10.1001/jamanetworkopen.2024.21285

**Published:** 2024-06-17

**Authors:** Carrie Henning-Smith, Teri Fritsma, Andrew P.J. Olson, Selam Woldegerima, Hannah MacDougall

**Affiliations:** 1 Division of Health Policy and Management, University of Minnesota School of Public Health, Minneapolis; 2 University of Minnesota Rural Health Research Center, Minneapolis; 3 Minnesota Department of Health, St Paul; 4 Division of Hospital Medicine, Department of Medicine; Division of Pediatric Hospital Medicine, Department of Pediatrics, University of Minnesota Medical School, Minneapolis; 5 Medical Education Outcomes Center, University of Minnesota Medical School, Minneapolis; 6 University of Minnesota School of Social Work, St Paul

## Abstract

This survey study assesses factors associated with mental health care professionals choosing to practice in rural locations.

## Introduction

Rural areas of the US have long-standing inequities in mental health outcomes and access to services, due partly to rural mental health workforce shortages.^1,2^ Health care professionals have multifaceted reasons for choosing rural practice; however, previous research has not differentiated by professional type and had relatively small sample sizes.^3,4^ Research exploring rural health care practice more broadly has found that the most salient factor associated with choosing rural practice is having grown up in a rural area, but that other significant factors include financial incentives, practice characteristics, and training experiences.^5^

This study uses data from licensed mental health professionals to ask 2 questions. First, what factors are associated with mental health professionals choosing to practice in rural areas? Second, do those factors differ by professional type? We hypothesize practice location considerations differ by rurality and professional type.

## Methods

This survey study was approved by the institutional review boards of the University of Minnesota and Minnesota Department of Health (MDH). The survey used in this study is mandated in Minnesota state statute and it is obligatory for respondents; however, MDH assures respondents that their responses are private and cannot be shared at the individual level. This study adheres to the Strengthening the Reporting of Observational Studies in Epidemiology (STROBE) reporting guideline.

This study uses data from Minnesota’s health licensing boards and the MDH Healthcare Workforce Survey of active health care professionals collected from February 7, 2022, through February 7, 2023. Our study population included 4 health care professional groups: mental health clinicians who prescribe medications (psychiatrists and advance practice registered nurses with a mental health focus), licensed mental health professionals (including licensed professional clinical counselors and licensed independent clinical social workers), licensed psychologists (LPs), and licensed alcohol and drug counselors (LADCs). MDH surveys all of these professionals at the time they renew their license; response rates range from 70% to 95%. To create the final dataset, we merged the health licensing board and MDH survey data. Information about variables and measurements is provided in the eMethods in Supplement 1.

We ran binary logistic regression models to estimate the associations of the independent variables with the dependent variable: rural practice location. We estimated separate adjusted models for each of the 4 different professional groups. All analyses were conducted in SPSS software version 29.0.0.0 (IBM) with 1-tailed hypothesis tests. Significance was set at *P* < .05. Data were analyzed from December 27, 2023, and February 22, 2024.

## Results

There were 8908 professionals (mean [SD] age, 46.7 [12.4] years; 6992 [79%] female) in this study. Most professionals were practicing in urban areas, with psychologists least likely to be practicing in rural areas (43 of 1142 psychologists [4%]) and LADCs most likely to practice in rural areas (128 of 1214 LADCs [10%]) (Table 1). Most rural professionals grew up in a small town or rural area, with the highest rates among LADCs (92 LADCs [75%]). Having autonomy in one’s practice was most commonly endorsed as a very important consideration when deciding where to work and practice (Table 1).

**Table 1.** Demographic Characteristics and Distributions on Independent and Dependent Variables

Variables	Professional type by location, No. (%)^a^							
	Prescribers		Mental Health Professionals		Psychologists		LADCs	
	Rural	Urban	Rural	Urban	Rural	Urban	Rural	Urban
Sample	33 (4)	778 (96)	323 (6)	5248 (94)	43 (4)	1142 (96)	128 (10)	1213 (90)
Age, mean (SD), y	49.7 (12.7)	50.3 (12.7)	45.1 (10.8)	44.7 (11.34)	55.6 (12.0)	54.4 (13.2)	47.8 (11.9)	45.3 (12.5)
Sex								
Male	9 (27)	333 (43)	28 (9)	844 (16)	13 (30)	333 (29)	37 (29)	319 (26)
Female	24 (73)	445 (57)	295 (91)	4404 (84)	30 (70)	809 (71)	91 (71)	894 (74)
Where did you grow up?								
Large metropolitan or surrounding	5 (15)	370 (50)	48 (16)	2185 (46)	9 (22)	507 (49)	13 (11)	507 (46)
Small city	9 (27)	153 (21)	47 (16)	983 (21)	3 (7)	221 (22)	18 (15)	223 (20)
Small town or rural area	19 (58)	213 (29)	206 (68)	1575 (33)	29 (71)	301 (29)	92 (75)	375 (34)
Scaled variables, mean (SD)^b^								
Family considerations	7.18 (2.46)	6.64 (3.07)	6.41 (3.32)	6.13 (3.49)	5.77 (3.31)	6.17 (3.43)	6.30 (3.18)	5.60 (3.43)
Lifestyle and area considerations	8.18 (2.34)	7.89 (3.11)	7.31 (3.39)	7.29 (3.59)	7.74 (2.95)	7.69 (3.47)	7.56 (2.96)	6.98 (3.47)
Ordinal variables^c^								
Having autonomy in my practice	24 (73)	412 (57)	128 (43)	2189 (47)	23 (56)	482 (47)	51 (42)	480 (44)
Having a broad scope of practice	9 (27)	242 (33)	72 (24)	1301 (28)	16 (39)	310 (30)	19 (16)	248 (23)
Loan forgiveness	10 (30)	107 (15)	82 (27)	638 (14)	13 (32)	158 (15)	17 (14)	158 (14)
Internship, clinical training, or residency	5 (15)	224 (31)	55 (18)	974 (21)	6 (15)	239 (23)	25 (20)	269 (25)
Educational program	8 (24)	112 (15)	45 (15)	524 (11)	4 (10)	75 (7)	23 (19)	154 (14)
Financial incentive	7 (21)	150 (21)	32 (11)	897 (19)	8 (20)	140 (14)	15 (12)	203 (19)
Ability to specialize	11 (33)	298 (41)	62 (21)	1087 (23)	5 (12)	305 (30)	23 (19)	225 (21)
Certain types of patients	7 (21)	211 (29)	59 (20)	1203 (25)	10 (24)	287 (28)	27 (22)	257 (23)

Abbreviation: LADC, licensed alcohol and drug counselor.

^a^ The total counts include professionals who were currently practicing in a position related to their license and did not have missing values on the dependent variable (urban or rural area of practice). The total counts may vary slightly from question to question because of item nonresponse.

^b^ Family considerations scale ranges from 1 to 9; area/lifestyle considerations scale ranges from 1 to 10. Higher scores indicate more importance placed on each consideration.

^c^ Data are given for respondents who rated each item as very important. Response options were very important, somewhat important, not important at all, and did not apply to me.

Adjusting for covariates, growing up in a rural area was consistently associated with rural practice across professions (prescribers: adjusted odds ratio [AOR], 2.17; 95% CI, 1.72-2.62; *P* < .001; mental health professionals: AOR, 2.34; 95% CI, 2.18-2.50; *P* < .001; LPs: AOR, 2.70; 95% CI, 2.27-3.13; *P* < .001; LADCs: AOR, 2.94; 95% CI, 2.65-3.22; *P* < .001) (Table 2). Other factor associations varied by professional group (Table 2). Having autonomy at work was associated with rural practice for prescribers (AOR, 2.12; 95% CI, 1.50-2.74, *P* = .02) and LPs (AOR, 1.86; 95% CI, 1.40-2.32; *P* = .01). Loan forgiveness was associated with rural practice for mental health professionals (AOR, 1.70; 95% CI, 1.58-1.82; *P* < .001) and LPs (AOR, 1.48; 95% CI, 1.15-1.81; *P* = .03).

**Table 2.** Adjusted Odds of Practicing in Rural Location

Variable	Prescribers		Mental health professionals		Psychologists		LADCs	
	AOR (95% CI)	*P* value	AOR (95% CI)	*P* value	AOR (95% CI)	*P* value	AOR (95% CI)	*P* value
Age, per 1-y increase	1.02 (0.99 to 1.05)	.38	1.01(1.00 to 1.03)	.02	1.02 (0.99 to 1.05)	.13	1.02 (1.00 to 1.04)	.03
Female sex (vs male)	0.49 (to 0.35 to 1.33)	.09	0.55 (0.12 to 0.98)	<.01	1.08 (0.34 to 1.82)	.84	1.16 (0.69 to 1.63)	.54
Grew up rural (vs not)	2.17 (1.72 to 2.62)	<.001	2.34 (2.18 to 2.50)	<.001	2.70 (2.27 to 3.13)	<.001	2.94 (2.65 to 3.22)	<.001
Family considerations^a^	1.04 (0.88 to 1.20)	.66	1.03 (0.98 to 1.08)	.26	0.87 (0.75 to 0.99)	.02	1.05 (0.97 to 1.13)	.23
Area considerations^a^	0.92 (0.75 to 1.09)	.36	0.98 (0.93 to 1.03)	.50	0.97 (0.83 to 1.11)	.70	1.04 (0.94 to 1.13)	.43
Being able to have autonomy at work^a^	2.12 (1.50 to 2.74)	.02	0.96 (0.79 to 1.12)	.60	1.86 (1.40 to 2.32)	.01	1.10 (0.84 to 1.36)	.46
Being able to have a broad scope of practice^a^	0.96 (0.45 to 1.47)	.87	0.90 (0.73 to 1.07)	.24	0.96 (0.51 to 1.41)	.85	0.90 (0.64 to 1.16)	.41
Loan forgiveness^a^	1.40 (1.01 to 1.79)	.09	1.70 (1.58 to 1.82)	<.001	1.48 (1.15 to 1.81)	.03	1.04 (0.82 to 1.26)	.75
An internship prepared me^a^	0.67 (0.31 to 1.03)	.03	1.00 (0.87 to 1.14)	.95	0.74 (0.40 to 1.08)	.08	1.01 (0.79 to 1.23)	.93
A formal educational program prepared me^a^	1.14 (0.71 to 1.57)	.56	1.08 (0.93 to 1.23)	.31	0.99 (0.58 to 1.40)	.99	1.14 (0.90 to 1.38)	.28
Financial incentive^a^	0.95 (0.52 to 1.38)	.80	0.73 (0.59 to 0.87)	<.001	1.36 (1.00 to 1.72)	.09	0.76 (0.53 to 0.99)	.02
Being able to specialize in certain types of care^a^	0.79 (0.32 to 1.26)	.33	1.11 (0.94 to 1.29)	.23	0.48 (0.03 to 0.93)	<.001	0.90 (0.61 to 1.20)	.50
Being able to work with certain types of patients^a^	0.83 (0.31 to 1.35)	.49	0.92 (0.74 to 1.11)	.38	1.36 (0.86 to 1.86)	.23	1.15 (0.87 to 1.43)	.33

Abbreviations: AOR, adjusted odds ratio; LADCs, licensed alcohol and drug counselors.

^a^ Measured per 1-unit increase in the importance level rated for the item.

## Discussion

This findings of this survey study suggest that policy interventions to address rural mental health workforce shortages should focus on bolstering the pathway of rural residents entering practice, but other efforts need to be tailored depending on professional type. Limitations of this study include that the data are from only 1 state and do not account for care delivered virtually (ie, telehealth). However, the variation we identified in factors associated with rural mental health care practice indicates a need for a targeted and multifaceted recruitment strategy.

## Notes

Supplement 1.
eMethods.

Supplement 2.
Data Sharing Statement
