# Supplementary material for: Decisions to Practice in Rural Areas Among Mental Health Care Professionals
Source: JAMA Netw Open. 2024 Jun 17;7(6):e2421285. doi: 10.1001/jamanetworkopen.2024.21285 (PMC11184455; doi:10.1001/jamanetworkopen.2024.21285)
Supplement: Supplement 1. — eMethods. [file jamanetwopen-e2421285-s001.pdf]

Supplemental Online Content

Henning-Smith C, Fritsma T, Olson APJ, Woldegerima S, MacDougall H. Decisions to practice in rural areas among mental health care practitioners. JAMA Netw Open. 2024;7(6):e2421285. doi:10.1001/jamanetworkopen.2024.21285

eMethods.

This supplemental material has been provided by the authors to give readers additional information about their work.

© 2024 Henning-Smith C et al. *JAMA Network Open.*

Supplemental Methods

Variables and Measurement

Dependent variable. The primary outcome is the choice to practice in a rural area, based on providers' practice address in 2023. We used Rural-Urban Commuting Area (RUCA) categories to assign addresses to urban (Urban and Micropolitan/Large Town) or rural (Small Town/Small Rural and Isolated Rural).

Covariates. We include a measure to control for having "grown up rural." The MDH survey asks: "*Which of these best describes the area in which you grew up?*" with response options: "a large metropolitan area or surrounding," "a small city," or "a small town or rural area." We operationalized rurality differently than the RUCA measure because cognitive survey testing showed that respondents do not consistently distinguish between "small town" and "isolated rural." We also include covariates for provider age and sex.

Independent variables. The final survey instrument included 16 unique items asking about considerations in choosing where to practice, representing five general categories: family considerations, practice considerations, financial and loan forgiveness incentives, education and training experiences, and characteristics of the area itself. We combined the family- and area-related considerations together into two separate scaled variables in order to simplify the interpretation of results. Please see the following question wording and Supplemental Table 1 for details about how these were asked and what items were included for each category.

Question wording: *Think back to how you made the decision to live in your general area. How important were each of the following considerations? [With response options: "Very important" | "Somewhat important"| "Not important at all" | "Did not apply to me"]*

© 2024 Henning-Smith C et al. *JAMA Network Open.*

Supplemental Table 1: Considerations in choosing where to practice survey items

Question	Category
The lifestyle in this area	Area considerations
Whether my partner/spouse would have job opportunities	Family considerations
Whether I would have autonomy in my work	Practice considerations
Whether this would be a good place to raise children	Family considerations
An internship, clinical training, or residency exposed me to what it's like to work in this area, or a similar one	Education and training characteristics
Living close to family or friends	Family considerations
Whether I would be able to work with certain types of patients or clients	Practice considerations
A financial incentive to live here, such as higher pay or a hiring bonus	Financial incentive
The quality of life in this area	Area considerations
Whether I could specialize in certain types of care in this area	Practice considerations
Whether I could (or did) receive a loan forgiveness award	Financial incentive
My educational program emphasized caring for patients/clients in an area like this (e.g., urban or rural)	Education and training characteristics
Whether or not I could find a romantic partner in this area	*Not included in model*
Whether I could have a broad scope of practice (variety) in my work	Practice considerations
Whether the community here would be a good fit for me	Area considerations
A "calling" to work with patients/clients in this type of area	Practice considerations

© 2024 Henning-Smith C et al. *JAMA Network Open.*
