# Supplementary material for: Decisions to Practice in Rural Areas Among Mental Health Care Professionals
Source: JAMA Netw Open. 2024 Jun 17;7(6):e2421285. doi: 10.1001/jamanetworkopen.2024.21285 (PMC11184455; doi:10.1001/jamanetworkopen.2024.21285)
Supplement: Supplement 2. — Data Sharing Statement [file jamanetwopen-e2421285-s002.pdf]

Data Sharing Statement

Henning-Smith. Decisions to Practice in Rural Areas Among Mental Health Care Professionals. *JAMA Netw Open*. Published June 17, 2024. doi:10.1001/jamanetworkopen.2024.21285

Data
Data available: No

Additional Information
Explanation for why data not available: Data are part of larger licensing process.
